# Nanoarchitectonics + future leaders = bright success in materials science and technology

**DOI:** 10.1088/1468-6996/16/1/010302

**Published:** 2015-02-25

**Authors:** Katsuhiko Ariga, Martin Pumera, Masakazu Aono

**Affiliations:** 1 World Premier International Center for Materials Nanoarchitectonics (WPI-MANA), National Institute for Materials Science (NIMS), Japan; 2 School of Physical and Mathematical Science, Division of Chemistry and Biological Chemistry, Nanyang Technological University, Singapore

Great progress requires great ideas. Over the past few decades probing the unusual behavior of nanoscale systems has been a great source of inspiration for developing device functionalities, as well as rethinking the way we understand the fundamental principles that govern them. However the same divergence from traditional behavior in nanoscale systems that has catalyzed these creative advances presents challenges since the old rules for fabrication and design do not always apply. Nanoarchitectonics is a new approach for nanoscale research that embraces the quirks of nanoscale systems to accelerate progress in the field.

Nanotechnology is essentially traditional technology with much smaller features. However, it is not always possible to simply miniaturize methodologies from microtechnology to the nanoscale. Phenomena at the nanoscale are highly influenced by thermal and statistical fluctuations, as well as mutual interactions between components. These effects and interactions have to be harmonized in a carefully designed architecture to create functional nanomaterials. This informed and innovative approach to handling the building blocks in nanotechnology is what we describe as nanoarchitectonics.

The concept of nanoarchitectonics represents a paradigm shift from traditional nanotechnology. It was first proposed by Masakazu Aono at the 1st International Symposium on Nanoarchitectonics Using Suprainteractions in 2000 in Tsukuba, Japan [1, 2]. Nanoarchitectonics constructs functional materials through coordinating atomic- and molecular-level control, chemical nanofabrication, self-assembly and field-controlled organization, as well as theoretical modeling. These working principles can be applied to all categories of materials, regardless of the material type, be that inorganic, organic, or biomaterials. Electroactive, photoactive and bioactive systems and their functional units can be combined into architectures that produce soft nanomaterials, coordination polymers, nanomachines, functionalized nanocarbons, catalytic materials, and more. The production of these materials may be driven by spontaneous molecule–molecule interactions, as in self-assembly, or external forces, such as laser irradiation.

The research described in this issue builds on many of these concepts and design features. It includes synthesis and functionalization of self-propelled motors, optoelectronic soft materials, carbon electrocatalysts for oxygen reduction, photoluminescent coordination polymers, photocatalytic nanodisks, structure-controlled perovskites, and shape-memory surfaces for cells. It also covers some of the fabrication techniques, such as the controllable assembly of nanoparticles by laser writing, platinum electrocatalyst production by arc plasma deposition, and wrapping carbon nanotubes in polymers.

Nanoarchitectonics provides the concept to catalyze the next leaps and bounds of progress in materials science and technology. All that remains is to encourage the talented scientists working with it. As a result, an important mission of this special issue is to highlight future leaders. It is our belief that ‘nanoarchitectonics + future leaders’ will lead to ‘bright success in materials science and technology’.
